# Barley Seed Aging: Genetics behind the Dry Elevated Pressure of Oxygen Aging and Moist Controlled Deterioration

**DOI:** 10.3389/fpls.2016.00388

**Published:** 2016-03-31

**Authors:** Manuela Nagel, Jan Kodde, Sibylle Pistrick, Martin Mascher, Andreas Börner, Steven P. C. Groot

**Affiliations:** ^1^Genebank Department, Leibniz Institute of Plant Genetics and Crop Plant Research (IPK Gatersleben)Stadt Seeland, Germany; ^2^Wageningen UR, Plant Research International B.V.Wageningen, Netherlands

**Keywords:** caryopsis, seed conservation, seed storage, genotype, germination, linkage mapping

## Abstract

Experimental seed aging approaches intend to mimic seed deterioration processes to achieve a storage interval reduction. Common methods apply higher seed moisture levels and temperatures. In contrast, the “elevated partial pressure of oxygen” (EPPO) approach treats dry seed stored at ambient temperatures with high oxygen pressure. To analyse the genetic background of seed longevity and the effects of seed aging under dry conditions, the EPPO approach was applied to the progeny of the Oregon Wolfe Barley (OWB) mapping population. In comparison to a non-treated control and a control high-pressure nitrogen treatment, EPPO stored seeds showed typical symptoms of aging with a significant reduction of normal seedlings, slower germination, and less total germination. Thereby, the parent Dom (“OWB-D”), carrying dominant alleles, is more sensitive to aging in comparison to the population mean and in most cases to the parent Rec (“OWB-R”), carrying recessive alleles. Quantitative trait locus (QTL) analyses using 2832 markers revealed 65 QTLs, including two major loci for seed vigor on 2H and 7H. QTLs for EPPO tolerance were detected on 3H, 4H, and 5H. An applied controlled deterioration (CD) treatment (aged at higher moisture level and temperature) revealed a tolerance QTL on 5H, indicating that the mechanism of seed deterioration differs in part between EPPO or CD conditions.

## Introduction

Knowledge of the mechanism of seed longevity has become important since international companies have started to ship seeds around the world and gene banks initiated the first seed collections at the beginning of the twentieth century. Thereby, long-term survival is greatly influenced by plant breeders who continuously work on seed quality performance.

Desiccation-tolerant (orthodox) seeds extend their life span when they reach maturity, dry, and enter the glassy state (Walters et al., 2005a; Buitink and Leprince, 2008). A glass is an amorphous, solid state with high viscosity (Buitink and Leprince, 2004) where metabolic processes are reduced to a minimum or can be even excluded (Kranner et al., 2010; Fernández-Marín et al., 2013). In this state, reactive oxygen species (ROS; Bailly, 2004; El-Maarouf-Bouteau et al., 2011), non-enzymatic Amadori and Maillard reactions (Sun and Leopold, 1995), and lipid peroxidations can damage macromolecules such as DNA, proteins, and lipids, and lead to seed deterioration. Further extrinsic factors can be multifaceted and include abiotic and biotic stress during seed development, seed maturity, harvest, drying, and cleaning. The rate of decay depends largely on the storage environment, as temperatures (Roberts, 1973), atmosphere (Groot et al., 2014) and relative humidity (RH) interact with the genotype (Walters et al., 2005b; Nagel and Börner, 2010). Consequently, a temperature or RH increase cause a viscosity decrease and thus a glass to rubber state change with higher molecular mobility and reaction kinetics (Walters, 1998).

The investigation of seed deterioration under natural conditions is protracted, as optimally stored orthodox seeds can survive for decades without any effect on the germination. To study seed deterioration more quickly, experimental approaches were invented by Roberts (1960) and refined by Hampton and Tekrony (1995) and Hay et al. (2008). Basically, temperatures are adjusted to above 30°C and seed moisture levels are increased, which converts the glassy state into a rubber state, with dramatic effects on chemical reactions (Walters, 1998). These methods have been widely applied for investigations on ecological (Probert et al., 2009), molecular (Waterworth et al., 2010), biochemical (Lehner et al., 2008), and genetic (Sasaki et al., 2015) levels. However, biochemical processes of artificially aged seeds in the rubber state can deviate from those in industrial dry storage at ambient temperatures or in cold, dry storage in gene banks, such as the half-reduction potential of the antioxidant glutathione and an assumed shift in pH (Nagel et al., 2015). Therefore, Groot et al. (2012) developed the “elevated partial pressure of oxygen” (EPPO) approach, which avoids a glass–rubber transition while increasing oxidation by exposing the dry seeds to high oxygen pressure (up to 18 MPa). This treatment affects germination in such a way that the visual appearance of seed deterioration is consistent with that observed after long term dry storage.

So far, the genetic backgrounds of seed longevity have been investigated by exposing seeds to relatively moist experimental aging conditions. Quantitative trait locus (QTL) analysis and association mapping revealed that longevity QTLs often co-located with QTLs for other traits, including morphological traits such as spike compactness (Rehman Arif et al., 2012). In barley, whose seeds are categorized as having medium-long storage potential (Walters et al., 2005b), QTLs for longevity co-located with QTLs for plant height and naked caryopsis on two chromosomes (Nagel et al., 2009).

The aim of this study was to investigate the genetic background of barley seed longevity by applying the EPPO treatment to dry-stored seeds. Double haploid (DH) lines of the Oregon Wolfe Barley (OWB) mapping population, genotyped with 2383 markers (Chutimanitsakun et al., 2011), were equilibrated to 40% RH at 20°C and exposed to high oxygen pressure at 18 MPa. Control and deterioration effects after treatment were assessed by germination speed and compared with previous experiments using CD (Nagel et al., 2009). Both data sets were analyzed and reanalyzed with the currently available marker sets and differences after QTL mapping are discussed.

## Material and methods

### Experimental material

Caryopses, henceforth termed seeds, of the DH OWB mapping population were studied on their ability to cope with stress during storage. The set of 94 spring barley lines was developed by pollinating the *Hordeum vulgare* F1 hybrid produced by the dominant parent Dom (“OWB-D”) × recessive parent Rec (“OWB-R”) with *H. bulbosum* (Costa et al., 2001). Characteristics of “OWB-D” and “OWB-R” were selected as dominant and recessive morphological marker stocks (Wolfe and Franckowiak, 1991), whereby more information can be found at http://barleyworld.org/oregonwolfe.php. A marker set of 2832 markers including 463 Restriction site Associated DNA (RAD) markers was developed by Chutimanitsakun et al. (2011) and is available at http://wheat.pw.usda.gov/ggpages/maps/OWB/. In 2005, a large number of seeds were produced under optimum conditions to maximize seed yield and quality in Gatersleben, Germany, and stored in conditions of 10% seed moisture content and −18°C.

### Storage treatment

The EPPO treatment was applied at Wageningen UR in 2011 according to Groot et al. (2012). Seeds of the OWB population were equilibrated to 40% RH at 20°C for 2 weeks. Thereafter, the seeds were stored under ambient atmospheric pressure in 1 l rubber-sealed glass jars (TC, treatment control) or in 12 l steel tanks under an elevated partial pressure of oxygen (EPPO) or nitrogen (EPPN) gas. The latter were used to control for potential damage induced by the high pressure and/or pressure release from the high pressure storage. The seed samples were placed within the tanks in 13 ml polystyrene tubes (Sarstedt, Germany) perforated with holes of ~1 mm diameter and closed with low-density polyethylene push caps. The tanks were filled slowly (~6 bar/min) with oxygen or nitrogen from large buffer tanks, until the tank pressure reached ~18 MPa. To minimize temperature changes during filling, the tanks were placed in water at ambient temperature. Filling of the tanks with oxygen was performed by skilled staff at a local scuba diving shop (4Divers, Veenendaal, The Netherlands). As tanks were not flushed before filling, they retained the initial atmospheric amounts of oxygen (0.021 MPa partial pressure), nitrogen and other minor gases present in air. Silica gel, equilibrated to 40% RH, was also added to the tanks and glass jars to buffer RH in the containers. The tank pressure was released with an average relative pressure decline of 0.5% per min (maximum 3.0%) using a computer-controlled relative flow rate. Between experiments and prior to germination testing, seeds were stored in a cabinet with air circulating above a saturated CaCl_2_ salt solution (RH 35%) at a temperature of 20°C. Seeds were packaged in sealed laminated foil bags and sent to IPK Gatersleben. As an initial test, 10 lines of the “W766” barley population, multiplied and stored together with seeds of the OWB population, were treated for 4, 6, 8, and 12 weeks in oxygen at 18 MPa (atmospheric pressure is ~0.1 MPa) and showed a significant reduction in germination and germination speed between 8 and 12 weeks (Supplementary Figure 1). For the main experiment, the seeds were stored for 9 weeks under EPPO, EPPN, or ambient pressure conditions (TC).

### Germination test

At IPK Gatersleben, seeds were subjected to germination tests. Four replicates of 50 seeds (for a few lines, only 10–40 seeds per replicate were available) were placed on moistened filter paper and kept in a germination chamber at 20°C, 60% RH for 8 h of light. Germinating seeds were determined daily by examining radicle protrusion. After a period of 10 days, seeds were classified into normal seedlings, which show the potential to develop into satisfactory plants; abnormal seedlings, which are damaged, deformed, or decayed, and do not show potential to develop into a normal plant; and non-germinating seeds according to (ISTA, 2012). The percentage of total germinated seeds (%TG) and normal seedlings (%NS), the time to reach 50% of the maximum germination (T50), and the area under the curve (AUC) after 100 h were calculated using the curve-fitting module of the Germinator (Joosen et al., 2010).

### Controlled deterioration

In 2008, DH lines of the OWB population were experimentally aged at 44°C and 18% seed moisture content, following the procedure of CD by Hampton and Tekrony (1995). Four replicates of 50 seeds [control of 2008 tests (C08) and controlled deteriorated seeds of 2008 tests (CD08)] were germinated according to ISTA (2008) and published in Nagel et al. (2009). Here, %NS and %TG were recalculated and, on the basis of viability equation *v* = *K*_*i*_–σ^−1^ p of Roberts (1973), the half-viability period (P50) for CD and EPPO aging was analyzed using %NS for initial seed germination (*K*_*i*_) and 63 days and 3 days to calculate sigma (σ) for EPPO and CD, respectively. Further, %NS were transformed to probit units and used to subtract control and EPPN from the EPPO and CD treatments and to overcome the effect of the different initial germinations of the genotypes. This resulted in additional traits for QTL mapping: 3-year storage (C08-TC), TC-EPPN, EPPO-TC, EPPO-EPPN, CD08–TC08.

The Shapiro–Wilk test indicated that, except for T50, all data did not match the pattern produced by a population with normal distribution. Therefore, Friedman Repeated Measures Analysis of Variance on Ranks, followed by Tukey's and Dunn's tests, was used to analyse differences (*P* < 0.05) between treatments.

### QTL mapping

QTL analyses for each of the phenotypic characters were conducted for the 93 OWB lines using the composite interval mapping procedure Zeng (1994) implemented in Windows QTL Cartographer 2.5 (Wang et al., 2011). Experiment-wise significance likelihood ratio test (LR) statistic thresholds (*P* ≤ 0.05) for QTL identification were determined with 1000 permutations, expressed as logarithm of odds (LOD = 0.217LR), and reinforced the set LOD threshold of 3. Significant QTL were characterized by the position of significant flanking markers, the marker with highest LOD, the proportion of phenotypic variance explained by the individual QTL (*R*^2^), the additive effect (expressed as one-half of the difference between the two allelic classes), and the LOD threshold. Negative values indicate that alleles were contributed by the parent “OWB-R” and positive values by the parent “OWB-D.”

### Gene content in QTL regions

Sequences of flanking markers of QTL regions were mapped to the whole-genome shotgun assembly of barley cv. “Morex” (The International Barley Genome Sequencing Consortium, 2012) with BWA mem version 0.7.12 (Li, 2013). Primary alignments with mapping quality ≥ 30 were extracted with SAMtools (Li et al., 2009), converted to BED format with BEDTools (Quinlan and Hall, 2010) and imported into R (R Core Team, 2015). Genetic positions of QTL regions in the POPSEQ genetic map (Mascher et al., 2013) were determined from the alignments of marker sequences to the Morex WGS contigs anchored by POPSEQ. If both flanking markers of a QTL regions were positioned in the POPSEQ map, the identifiers and functional annotation of all genes (The International Barley Genome Sequencing Consortium, 2012) between flanking markers were exported into a spreadsheet using functionalities of the R packages “openxlsx” (https://cran.r-project.org/web/packages/openxlsx/) and “data.table” (https://cran.r-project.org/web/packages/data.table/).

## Results

### Phenotypic data

**Figure 1:**
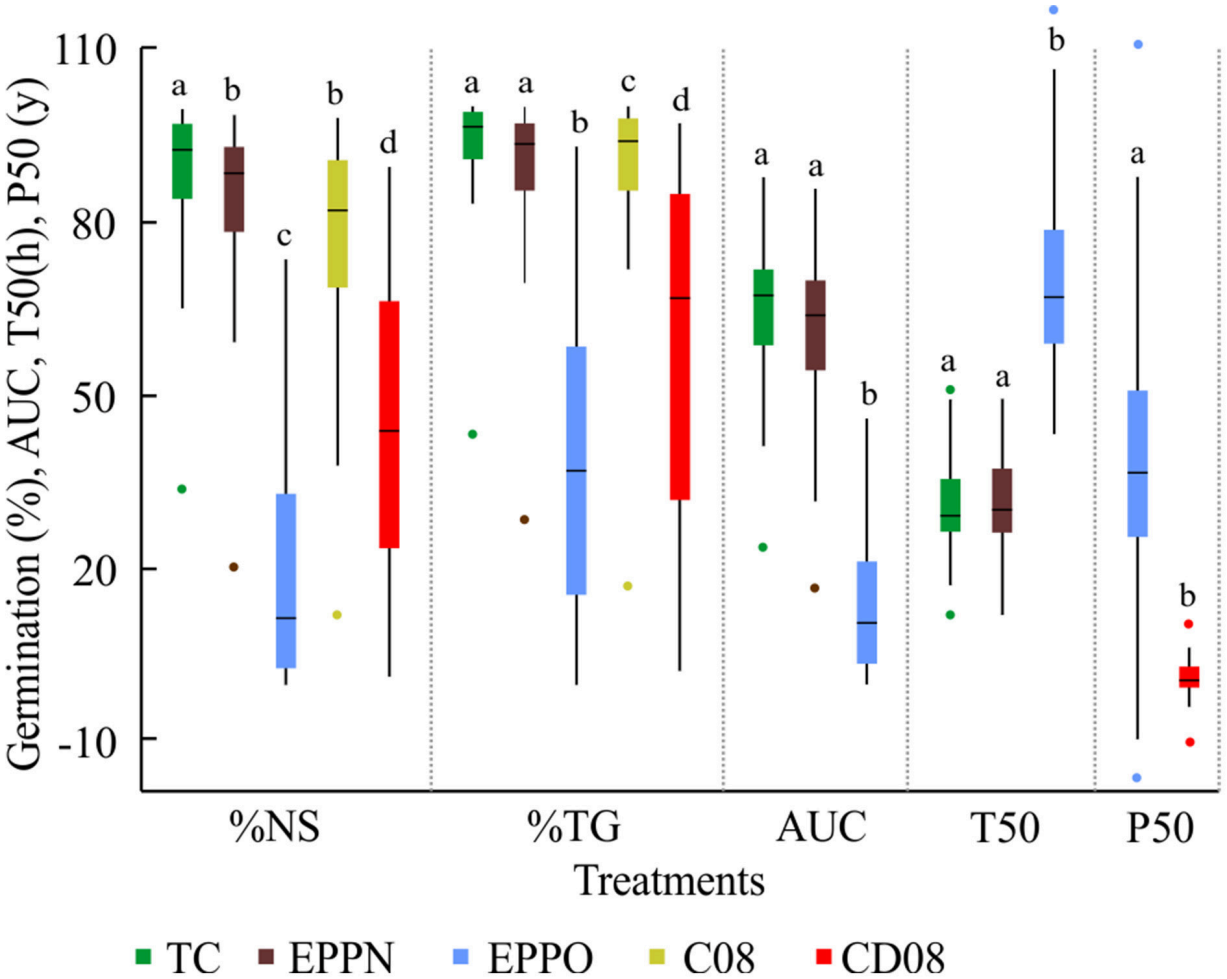
**Germination performance of 94 Oregon Wolfe Barley (OWB) lines after different storage treatments tested in 2008 and 2011**. Germination performance is expressed as the percentage of normal seedlings (NS%), total germination (TG%), area under the curve (***AUC***), time to 50% germination (T50 in hours), and half-viability period (P50 in days). Boxplots show minimum and maximum values (spots), 25% and 75% quartiles, and whiskers of the 1.5 interquartile range. Color code is given in the figure. C08, control performed in 2008; CD08, controlled deterioration performed in 2008; EPPN, elevated partial pressure of nitrogen storage; EPPO, elevated partial pressure of oxygen storage; TC, treatment control at ambient air pressure. a, b, c, and d symbolize significant differences between treatments at *P* < *0.05*.

Population lines were selected as dominant and recessive morphological marker stocks and show high phenotypic diversity. After 6 years of storage at 10% seed moisture content and −18°C, the seed material had on average 86.2 ± 16.3%NS and differed significantly between “OWB-D” (54.0%) and “OWB-R” (92.5%; Supplementary Figure 2). These differences were also reflected in the AUC and T50, which showed lower area and longer time to reach 50% germination for “OWB-D” (AUC = 35.8; T50 = 49.2 h) in comparison to “OWB-R” (AUC = 72.3; T50 = 27.6 h; Figure 1 and Supplementary Table 1). Except for T50 after EPPO and for %TG and P50 after CD08, the parent “OWB-D” showed worse performance than the mean of the population. “OWB-R” was only inferior to the mean of population after EPPO treatment.

**Figure 2:**
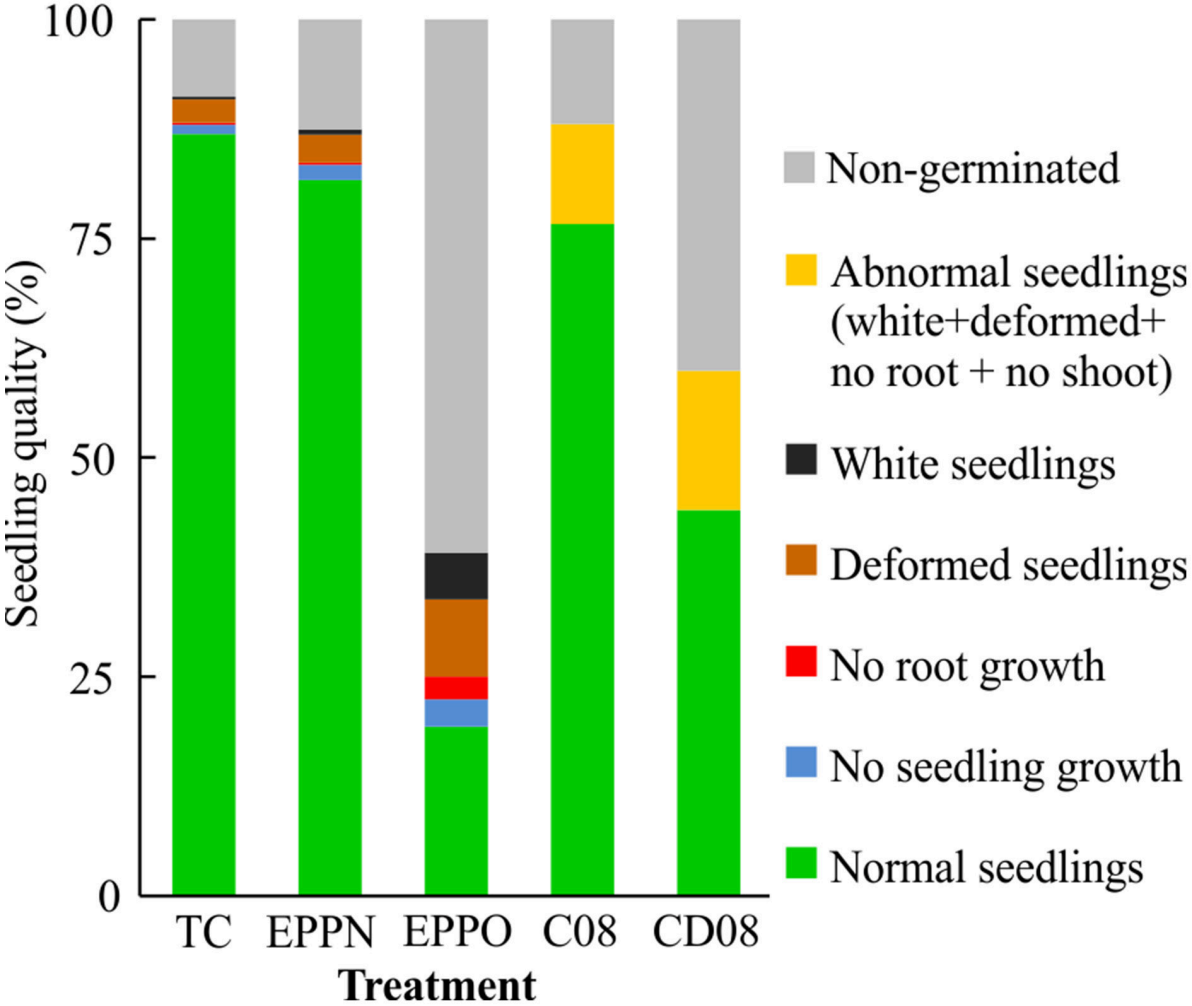
**Seedling quality of Oregon Wolfe Barley (OWB) lines after storage under atmospheric pressure (TC, treatment control), high-pressure nitrogen (EPPN), and high-pressure oxygen (EPPO)**. For comparisons, normal, abnormal (not discriminated into white, deformed, no root, no seedling growth), and non-germinated seedlings of C08 (control performed in 2008) and CD08 (controlled deterioration performed in 2008) are shown.

To compensate for potential damage induced by high pressure and pressure release, high nitrogen pressure (EPPN) was applied to seeds of the OWB population. The %NS was the only trait that showed a significant (*P* < 0.05) effect between both controls, storage at ambient pressure and EPPN treatment (Supplementary Table 1). In contrast, a high oxygen concentration, as applied in the EPPO treatment, affected germination significantly (*P* < 0.05), resulting in a reduced %NS, %TG, and AUC and extended T50 (Figure 1). Seedling quality differed between the treatments and was best in the control. After the high-pressure nitrogen treatment, there was a minor relative increase in abnormal seedlings with no shoot, white shoots, or deformations, and of non-germinated seeds, resulting in an ~5% decline in normal seedlings in comparison to the control seeds stored under ambient pressure conditions (Figure 2). The EPPO treatment caused extensive damage which resulted in a relative increase in seedlings with no shoot, no root, white shoots, deformation, and no germination of 190, 1026, 1600, 235, and 587%, respectively.

Comparing the 9 weeks EPPO treatment with the CD08 treatment, less damage was observed after the CD08 treatment. Only 38% more abnormal seedlings (not differentiated between white, deformed, no root and no shoot seedlings) were Counted (Figure 2). After 3 days of CD, CD08 was significantly different (*P* < 0.05) to control in 2008 and to EPPO treatment in 2011, and revealed an average decline in %NS and %TG of OWB population to 44.3 and 60.3%, respectively. Again, with some exceptions, the parent “OWB-D” performs less well than the population mean in all traits whereas “OWB-R” revealed higher initial germination but comparable germinations after CD.

On the basis of %NS in the ambient control, EPPN, and after EPPO, the P50 was calculated and resulted in a mean for the whole population of 38.5 ± 21.8 days after the EPPO treatment and of 2.3 ± 3.5 days after CD08 treatment. As demonstrated by the previous data, the parent “OWB-D” has a shorter half-viability period in comparison with “OWB-R” and the mean half-viability period of the DH population was higher than that of either parent.

Correlation analysis revealed that EPPN treatment showed a higher correlation with control treatments than did EPPO treatment. Here, the highest coefficients (*r* = −0.62; *P* < 0.001) were found between %TG of EPPO and T50 of controls, and indicate a weak relationship between both. All the germination parameters measured showed highly significant correlations (*P* < 0.001) with controls following EPPN treatment but this was not so following EPPO treatment, when not all parameters correlated significantly and those that did had lower r values (Table 1).

**Table 1:** **Correlation coefficients between germination parameters of different treatments**.

	**TC_%TG**	**TC_T50**	**TC_AUC**	**EPPN_%NS**	**EPPN_%TG**	**EPPN_T50**	**EPPN_AUC**	**EPPO_%NS**	**EPPO_%TG**	**EPPO_T50**	**EPPO_AUC**	**C08_%NS**	**C08_%TG**	**CD08_%NS**	**CD08_%TG**	**P50_EPPO**	**P50_CD08**
**TC_%NS**	0.93	−0.61	0.80	0.79	0.75	−0.54	0.70	0.38	0.52	−0.24	0.45	0.31	0.35		0.25	0.58	0.23
**TC_%TG**		−0.59	0.81	0.72	0.71	−0.54	0.67	0.33	0.48	−0.21	0.40	0.30	0.34		0.22	0.55	0.21
**TC_T50**			−0.93	−0.58	−0.58	0.84	−0.79	−0.34	−0.62	0.32	−0.56					−0.66	
**TC_AUC**				0.70	0.68	−0.81	0.84	0.33	0.60	−0.26	0.52		0.22			0.66	0.03
**EPPN_%NS**					0.95	−0.62	0.85	0.49	0.68		0.58	0.33	0.35			0.69	0.22
**EPPN_%TG**						−0.59	0.84	0.49	0.67	−0.23	0.59	0.29	0.31			0.70	0.24
**EPPN_T50**							−0.90	−0.24	−0.56	0.21	−0.46	−0.15	−0.19	0.22		−0.59	
**EPPN_AUC**								0.39	0.68	−0.23	0.58	0.23	0.26			0.71	
**EPPO_%NS**									0.85	−0.71	0.90	0.39	0.46	0.29	0.37	0.81	0.37
**EPPO_%TG**										−0.57	0.96	0.29	0.38			0.97	
**EPPO_T50**											−0.75	−0.21	−0.25		−0.24	−0.56	
**EPPO_AUC**												0.27	0.35			0.93	
**C08_%NS**													0.93	0.51	0.56	0.33	0.51
**C08_%TG**														0.59	0.66	0.40	0.57
**CD08_%NS**															0.97	0.07	0.83
**CD08_TG%**																	0.83
**P50_TC**																	
**P50_EPPN**																	
**P50_EPPO**																	
**P50_C08**																	

Spearmen correlation was performed on the basis of 93 Oregon Wolfe Barley (OWB) lines. C08, control performed in 2008; CD08, controlled deterioration performed in 2008; EPPN, elevated partial pressure of nitrogen storage; EPPO, elevated partial pressure of oxygen storage; P50, half-viability period; TC, treatment control at ambient air pressure; %NS, percentage of normal seedlings; %TG, percentage of total germination; T50, time to reach 50% of germinated seeds (hours); AUC, area under the curve; P< 0.001; P < 0.01; P < 0.05.

### QTL mapping

**Figure 3:**
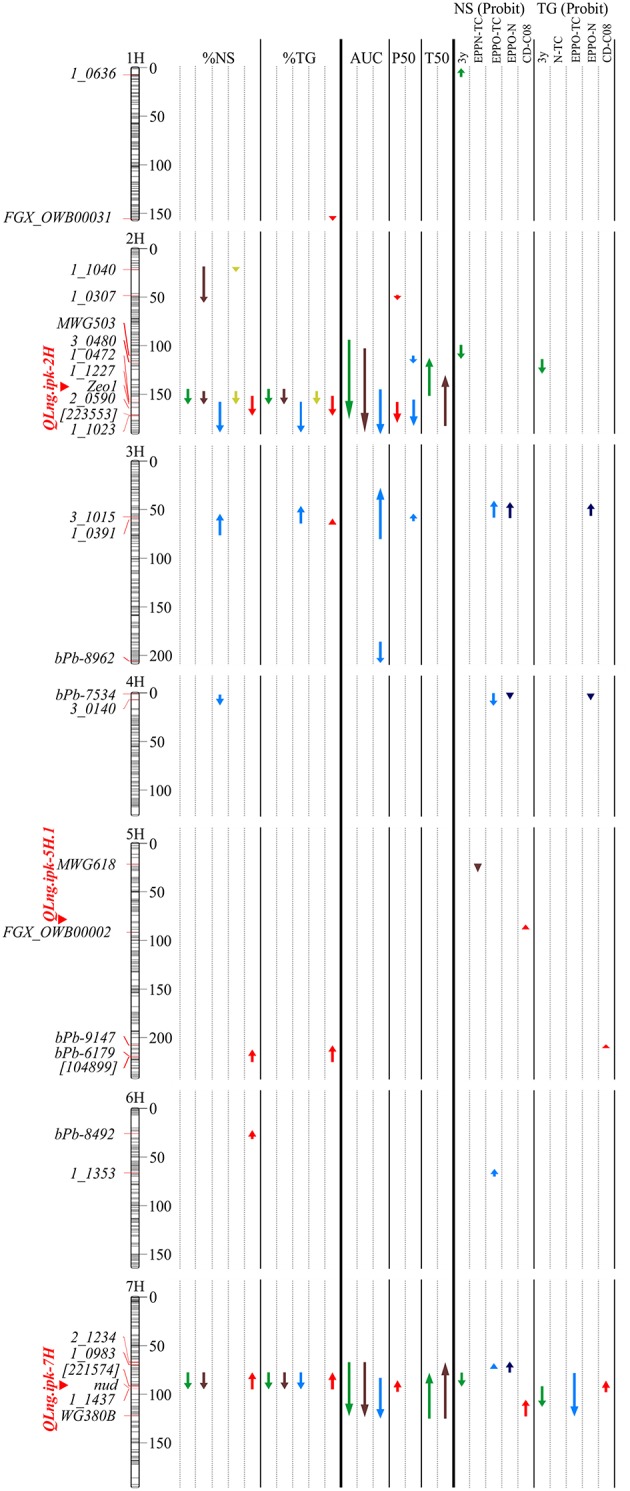
**Linkage map of Oregon Wolfe Barley (OWB) mapping population and quantitative trait locus (QTL) locations**. QTLs detected under control conditions are marked in green (TC, treatment control, dark green; C08, control prepared in 2008, light green), those under nitrogen pressure (EPPN) in brown, under oxygen pressure (EPPO) in blue, and those after CD in 2008 red. Map distances are shown in cM. QTLs declared above a logarithm of odds (LOD) threshold of 3. Arrow lengths indicate QTL intervals having LOD > 3 and arrows up indicate that alleles came from the “OWB-D” parent and those down from the “OWB-R” parent.

In total, 2832 markers were used to analyse the EPPO experiments and to reanalyse the results of the 2008 experiments (Figure 3; Table 2). In total, 65 significant QTLs (LOD > 3) were found across all chromosomes. Eight QTLs were detected under control, 9 for EPPN, 13 for EPPO conditions, 3 for C08, and 12 for CD08. In addition, 13 QTLs and 7 QTLs appeared for NS (probit) and TG (probit), respectively, after subtraction of control and EPPN treatment from EPPO, CD08, and C08. Most QTLs were found on chromosome 2H between 109 and 182 cM and on chromosome 7H between 63 and 122 cM. QTLs with the highest LOD scores were found for control (T50; *R*^2^ = 42.6; LOD = 17.7) on 7H, for EPPN (T50; *R*^2^ = 55.0; LOD = 25.1) on 7H, for EPPO treatment (AUC; *R*^2^ = 25.5; LOD = 11.5) on 3H, for C08 (%TG; *R*^2^ = 17.9; LOD = 7.3) on 2H, and for CD08 (%TG; *R*^2^ = 42.9; LOD = 16.3) on 2H.

**Table 2:** **Quantitative trait locus (QTL) analysis of the germination performance of the Oregon Wolfe Barley (OWB) mapping population after experimental aging methods**.

**Trait**	**Treat**	**Unit**	**C**	**Flanking markers**	**Marker**	**Position**	**LOD**	**R^2^**	**Additive effect**
NS	TC	%	2	146.7-164.3	1_0472	155.5	12.1	24.8	−893.1
	TC	%	7	82.6-94.7	1_1437	94.7	6.9	14.3	−614.3
	EPPN	%	2	21.9-55.2	1_0307	49.7	3.7	5.9	−488.5
	EPPN	%	2	148.9-164.3	Zeo1	159.9	12.9	26.8	−1096.1
	EPPN	%	7	82.6-94.7	nud	93.7	11.5	22.8	−914.7
	EPPO	%	2	159.9-182.2	1_1023	172.3	9.1	22.9	−946.6
	EPPO	%	3	55.2-69.4	1_0391	60.6	9.7	27.2	1027.7
	EPPO	%	4	1.1-9	3_0140	9.0	3.7	8.4	−586.1
	C08	%	2	−	1_1040	21.9	3.5	10.1	−855.7
	C08	%	2	148.9-164.3	1_1227	157.7	7.3	19.0	−1438.2
	CD08	%	2	152.2-174.5	2_0590	164.3	12.0	31.0	−1599.1
	CD08	%	5	212.1-228.6	bPb-6179	218.7	4.6	9.7	851.8
	CD08	%	6	20-30	bPb-8492	25.5	3.7	7.7	779.4
	CD08	%	7	82.6-94.7	1_1437	94.7	6.0	13.2	1033.7
NS	TC	Probit	2	86.9-105.6	1_0859	96.8	4.9	9.0	−23.1
	TC	Probit	2	146.7-164.3	1_0472	155.5	14.2	31.2	−42.7
	TC	Probit	7	87.1-94.7	1_1437	94.7	6.3	13.0	−26.1
	EPPN	Probit	2	12.1-17.5	3_0155	15.3	3.4	5.5	−16.3
	EPPN	Probit	2	148.9-164.3	Zeo1	159.9	17.1	40.0	−45.0
	EPPN	Probit	7	82.6-94.7	1_1437	94.7	8.9	16.7	−29.0
	EPPO	Probit	2	159.9-182.2	1_1023	172.3	9.7	22.1	−46.8
	EPPO	Probit	3	46.2-66.1	3_1015	57.3	10.2	23.8	46.5
	EPPO	Probit	4	0-9	bPb-7534	1.1	4.3	8.5	−29.0
	C08	Probit	2	146.7-164.3	3_0396	158.8	10.5	32.2	−40.2
	C08	Probit	5	211-220.9	[104899]	219.8	4.4	11.5	23.1
	CD08	Probit	2	152.2-174.5	2_0590	164.3	6.3	17.6	−33.1
	CD08	Probit	5	207.7-228.6	[104899]	219.8	7.4	20.5	34.6
NS	TC-C08	Probit	1	0-8.9	1_0636	8.9	3.2	9.2	24.4
	TC-C08	Probit	2	99-114.4	MWG503	113.3	4.8	13.5	−29.7
	TC-C08	Probit	7	82.6-94.7	[221574]	91.5	5.1	15.2	−31.0
	CD-C08	Probit	5	91.6-94.9	FGX_OWB00002	94.9	3.6	12.4	−38.6
	CD-C08	Probit	7	105.5-122	WG380B	122.0	6.5	25.7	54.3
	EPPO-TC	Probit	3	48.4-66.1	3_1015	57.3	7.3	17.4	44.6
	EPPO-TC	Probit	4	9-22.7	3_0140	9.0	3.4	7.4	−26.8
	EPPO-TC	Probit	6	59.7-69.5	1_1353	66.2	4.1	8.9	28.9
	EPPO-TC	Probit	7	73.8-74.9	2_1234	73.8	3.5	7.4	26.3
	EPPO-EPPN	Probit	3	46.2-66.1	3_1015	57.3	14.1	35.9	53.7
	EPPO-EPPN	Probit	4	0-1.1	bPb-7534	1.1	3.7	7.0	−25.0
	EPPO-EPPN	Probit	7	67.3-82.6	1_0983	74.9	4.9	10.1	27.8
	EPPN-TC	Probit	5	21.4-23.6	MWG618	21.4	3.6	13.8	−23.9
TG	TC	%	2	146.7-164.3	1_0472	155.5	12.1	30.3	−821.9
	TC	%	7	82.6-94.7	1_1437	94.7	6.1	13.8	−508.0
	EPPN	%	2	146.7-164.3	1_0472	155.5	12.3	28.5	−977.6
	EPPN	%	7	82.6-94.7	1_1437	94.7	9.4	19.9	−760.4
	EPPO	%	2	159.9-182.2	1_1023	172.3	8.6	18.5	−1226.8
	EPPO	%	3	46.2-65	3_1015	57.3	6.8	13.9	1031.1
	EPPO	%	7	82.6-94.7	1_1437	94.7	9.2	20.8	−1290.1
	C08	%	2	148.9-164.3	1_1227	157.7	7.3	17.9	−1420.9
	CD08	%	1	−	FGX_OWB00031	155.1	3.6	7.0	−886.0
	CD08	%	2	152.2-174.5	2_0590	164.3	16.3	43.0	−2236.5
	CD08	%	3	56.2-58.4	3_1015	57.3	3.8	7.1	882.4
	CD08	%	5	207.7-228.6	[104899]	219.8	8.2	17.3	1356.4
	CD08	%	7	82.6-94.7	1_1437	94.7	3.9	6.1	852.9
TG	TC	Probit	2	91.3-105.6	1_0859	96.8	4.2	8.4	−24.5
	TC	Probit	2	146.7-164.3	1_0472	155.5	11.7	27.3	−44.2
	TC	Probit	6	−	3_1308	45.4	3.0	5.9	19.9
	TC	Probit	7	94.7-108.8	1_1437	94.7	5.2	11.4	−26.9
	EPPN	Probit	2	93.5-113.3	MWG503	113.3	4.4	7.2	−22.0
	EPPN	Probit	2	148.9-164.3	Zeo1	159.9	13.5	28.5	−41.9
	EPPN	Probit	6	64-69.5	bPb-9082	69.5	4.1	6.8	21.8
	EPPN	Probit	7	82.6-94.7	nud	93.7	11.4	22.6	−35.7
	EPPO	Probit	2	159.9-173.4	3_0248	163.3	12.7	34.3	−56.1
	EPPO	Probit	3	35.1-55.2	FGX_OWB00221	46.2	4.5	9.5	29.4
	EPPO	Probit	7	82.6-94.7	nud	93.7	8.1	18.6	−41.2
	C08	Probit	2	146.7-182.4	3_0396	158.8	11.9	35.4	−50.6
	C08	Probit	5	181.3-182.4	[105067]	182.4	3.3	7.9	23.0
	CD08	Probit	2	152.2-174.5	2_0590	164.3	12.6	32.2	−55.1
	CD08	Probit	5	207.7-228.6	bPb-6179	218.7	6.7	14.3	35.2
	CD08	Probit	6	20-31.1	bPb-8492	25.5	4.0	8.3	27.0
	CD08	Probit	7	91.5-94.7	1_1437	94.7	3.7	7.5	25.8
TG	3y	Probit	2	113.3-127.6	3_0480	121.0	5.5	16.6	−37.2
	3y	Probit	7	94.7-108.8	1_1437	94.7	4.4	13.8	−33.5
	CD-C08	Probit	5	−	bPb-9147	207.7	3.2	11.6	25.9
	CD-C08	Probit	7	94.7-122	1_1437	94.7	3.5	13.9	27.2
	EPPO-TC	Probit	3	47.3-66.1	3_1015	57.3	5.6	17.7	35.9
	EPPO-EPPN	Probit	3	48.4-66.1	3_1015	57.3	8.0	24.5	53.9
	EPPO-EPPN	Probit	4	0-9	bPb-7534	1.1	3.9	11.0	−23.3
AUC	TC	mm^2^	2	91.3-173.4	1_0472	155.5	7.5	12.8	−5.6
	TC	mm^2^	7	63.9-119.8	1_1437	94.7	13.0	26.5	−7.4
	EPPN	mm^2^	2	100.1-186.6	Zeo1	159.9	14.3	21.3	−8.2
	EPPN	mm^2^	7	63.9-120.9	nud	93.7	20.3	36.8	−9.7
	EPPO	mm^2^	2	142.3-188.8	Zeo1	159.9	10.2	21.6	−5.7
	EPPO	mm^2^	3	27.5-80.3	1_0391	60.6	11.5	25.5	6.2
	EPPO	mm^2^	3	185.9-206.7	bPb-8962	206.7	3.7	6.8	−3.3
	EPPO	mm^2^	7	80.4-122.0	nud	93.7	5.6	10.4	−4.0
T50	TC	h	2	109.9-148.9	FGX_OWB00464	127.6	6.5	13.9	3.0
	TC	h	7	63.9-118.7	nud	93.7	17.7	42.6	5.0
	EPPN	h	2	127.6-180.0	Zeo1	159.9	7.0	9.3	2.5
	EPPN	h	7	63.9-122.0	1_1437	94.7	25.1	55.0	5.6
P50	EPPO	d	2	109.9-115.4	FGX_OWB00273	109.9	4.1	9.7	−7.9
	EPPO	d	2	159.9 – 180.0	1_1023	172.3	9.2	26.1	−12.7
	EPPO	d	3		1_0391	60.6	3.2	7.2	6.4
	CD	d	2	49.7-50.8	1_0307	164.3	3.1	10.4	−1.0
	CD	d	2	164.3-176.7	[223553]	171.3	4.2	12.4	−1.2
	CD	d	7	80.4-94.8	[221574]	91.5	7.2	23.3	1.7

QTLs declared above a logarithm of odds (LOD) of 3. Positive additive effects indicate a contribution by “OWB-D.” C08, control performed in 2008; CD08, controlled deterioration performed in 2008; EPPN, elevated partial pressure of nitrogen storage; EPPO, elevated partial pressure of oxygen storage; %NS, percentage of normal seedlings; %TG, percentage of total germination; TC, treatment control at ambient air pressure; T50, time to reach 50% of germinated seeds (hours); AUC, area under the curve; P50, half-viability period. Chr, chromosome; R^2^, proportion of explained phenotypic variance (%); cM, centiMorgan. –, before the additive effect of the allele indicates that the larger value allele came from the “OWB-R”; +, before the additive effect of the allele indicates that the larger value allele came from the “OWB-D” parent.

Subtraction of control or EPPN treatment from EPPO, CD, and C08 is assumed to compensate for the effect of control and high-pressure treatment and reveal treatment-specific loci. QTLs specific for EPPO were found on 3H, 4H, and 6H. On 5H, two QTLs were found for CD (94.9 and 207.7 cM) and one for EPPN (21.4 cM) and, on 2H, one QTL accounted for the differences after a 3-year storage period. Between 67 and 122 cM on chromosome 7H, QTLs were detected under all treatments.

Interestingly, alleles with the greatest effect on most QTLs (39 out of 65) were contributed by the parent “OWB-R” showing the highest additive effects in CD08 and EPPO treatment with −2236.5 and −1290.1 for %TG. The dominant parent “OWB-D” contributed the most alleles for QTLs on chromosome 3H after EPPO treatment and on 5H and 7H after CD08 treatment.

## Discussion

### High nitrogen pressure induce white seedling appearance

Anoxic conditions (produced by storage under 100% nitrogen gas) are used in the food industry to preserve organic material from microbial growth (Van Campenhout et al., 2013). In the current experiment, EPPN was used as a second control to compensate for the potential effects of high pressure and pressure release. With respect to %TG, AUC, and T50, high nitrogen pressure did not induce a significant effect compared to the control under ambient air pressure, but %NS was significantly reduced, as a higher number of seedlings with white shoots and no roots were produced. A release of pressure from the tanks at the end of the storage period can create an increase in the volume of gases trapped in internal spaces, thereby causing physical disruption of tissues and producing seedlings with deformations. However, in our experiments with barley, the frequency of seedlings with deformations was not increased. A possible explanation for the white seedlings is nitrogen conversion to nitrogen oxides, which affect metabolism, oxidation state, or signaling (Bethke et al., 2007). This might also explain the observed dormancy alleviation for some lines, as indicated by an increase in the %NS.

### Detrimental effects of EPPO on seed germination

Oxygen is essential for the survival of most living organisms under moist conditions, as it is required for aerobic respiration. With desiccation tolerant organisms such as seeds, respiration activity ceases under dry conditions and they can survive without oxygen. Molecular oxygen can have a detrimental effect through the formation of super oxide which reacts with almost all organic components and damages DNA, proteins and lipids, resulting in membrane leakage and cell death (Van Breusegem and Dat, 2006). The effect of high oxygen pressure was investigated by Caldwell (1964), who found that dry bean and pea seeds are able to tolerate high atmospheric oxygen pressure whereas imbibed beans and peas cannot. In our experiments, the dry barley seeds under investigation responded to high-pressure oxygen treatment with a considerable reduction in germination performance. After 9 weeks of dry storage under ~18 MPa oxygen, the %NS and %TG were reduced by 67 and 52% and the time to 50% germination almost doubled. This increased deterioration confirms the deleterious effects of oxygen on seeds.

### Aging treatment affects seed deterioration

The reduction in total number of germinating seeds indicates that barley seeds deteriorate more after 3 days of CD treatment than after 63 days of EPPO treatment. At the higher moisture content used in the CD, the fluidity of intercellular glasses increases (Walters et al., 2010) and biochemical processes are accelerated (Lehner et al., 2008). The EPPO approach intends to use seed equilibrated to ambient moisture and temperature conditions (here 20°C and 40% RH) and are assumed, according to Walters et al. (2005a), in the glassy state. As the glass-phase transition temperature is assumed to be hardly affected by higher gas pressure (Groot et al., 2012), seeds might remain in the glassy, metabolically inactive (Fernández-Marín et al., 2013) state during EPPO storage and deterioration is caused by oxygen. Groot et al. (2012) demonstrated that the EPPO approach might simulate ambient storage better than CD, as tocopherol content was linearly reduced with EPPO storage time, but not with CD, when seed quality declined.

### Linked markers and genes indicate relationship between morphology, stress response mechanism, and seed deterioration

In general, seed deterioration is affected by many factors, which pop up during seed development, ripening, threshing, cleaning and storage. This activates a plethora of mechanisms on cellular level as exemplified by the functional annotation of genes (Supplementary Table 2). However, most QTLs were found with two previously defined areas termed *QLng.ipk-2H* and *QLng.ipk-7H* (Nagel et al., 2009; Figure 3) and confirmed an association with the genetic panel of 175 diverse barley genotypes (Nagel et al., 2015). The QTL area on 2H estimated between 110 and 172 cM influences 21 traits, including plant height, spike length, grain number and grain yield. Thereby, the parent “OWB-D” provides dominant alleles to the progeny, is characterized by shorter plants (Chutimanitsakun et al., 2011) and is more sensitive to seed deterioration as “OWB-R”. In agreement with this the detected *Zeo1* gene determines plant height, spike compactness and kernel width (Franckowiak et al., 1997) caused by polymorphisms in the microRNA172 (miR172)-binding site of the barley ortholog of an APELATA2 transcription factor (HvAP2). It is indicated that changes result in altered timing of developmental events (Houston et al., 2013) which might affect seed quality and deterioration behavior. Further, in the centromeric region of 7H from 73 to 95 cM, 18 QTLs were identified. The *nud* gene, an ethylene response family transcription factor gene responsible for naked/covered caryopsis (Franckowiak and Konishi, 1997) is also in this area. It further controls the lipid biosynthesis pathway in the inner side of the hull, generating the adhesion between lemma and palea (Taketa et al., 2008) and β-glucan content (Capo-Chichi et al., 2012).

Further annotation of gene functions in the QTL areas revealed the appearance of harpin-induced protein 1 family (Gechev et al., 2004) and glutaredoxins (Rouhier et al., 2008) which indicate a relationship to abiotic and especially oxidative stress response mechanism and programmed cell death. Similar, the regulation of plant defense response against pathogens are demonstrated by the protein cysteine proteinase RD21a (Shindo et al., 2012), proline extensin-like receptor kinase 1 (PERK1; Silva and Goring, 2002), the disease resistance protein NBS-LRR (Belkhadir et al., 2004) and the MAP kinase substrate (MKS1; Andreasson et al., 2005). Due to metabolic inactivation during storage we speculate that proteins, kinases and factors are activated during seed development or germination.

### Specialized QTLs for tolerance to the two aging treatments

Germination after aging/storage is influenced by the initial germination which seeds show after harvest (Roberts, 1973). This effect is due to differences in susceptibility to pathogens, attraction for insects, and abiotic stress tolerances during seed production. Here, the effect of initial germination was mathematically omitted by the subtraction of control or EPPN from EPPO. This mathematical procedure elucidated and/or confirmed QTLs for EPPO tolerance on 3H, 4H, and 6H, for CD08 on 5H, and dormancy release on 2H calculated by differences between control in 2008 and the treatment control in 2011 (3 years). The QTL for CD08 shows co-linearity (Stein et al., 2007) to strong and highly significant QTLs found for rice seed deterioration (Sasaki et al., 2005, 2015). This region contains a gene, annotated as encoding trehalose-6-phosphate phosphatase, which can be used to manipulate abiotic stress tolerance in rice (Sasaki et al., 2005).

Biochemical pathways and deterioration processes are influenced by water activity (seed moisture level), temperature and atmosphere. In agreement with this, we observed QTLs in response to both EPPO and CD storage, as well QTLs expressed as a results of genetic variation in longevity under either EPPO or CD storage conditions. This indicates the causes of deterioration under CD and storage under EPPO are partly similar and partly distinct controlled.

## Author contributions

MN, AB, and SG conceived and designed research. MN, JK, SP, and SG conducted experiments. MN and SG analyzed data. MN and SG wrote the manuscript. All authors read and approved the manuscript.

## Funding

Financial support was provided by the EU “Seventh Framework Programme” grant EcoSeed (No. 311840) and the Netherlands Ministry of Economic Affairs in the research project Improved seed storage, in the frame of the “Topconsortium Kennis en Innovatie Tuinbouw & Uitgangsmaterialen program Meer met minder.” The publication of this article was funded by the Open Access fund of the Leibniz Association.

### Conflict of interest statement

The authors declare that the research was conducted in the absence of any commercial or financial relationships that could be construed as a potential conflict of interest. The reviewer PK and handling Editor declared their shared affiliation, and the handling Editor states that the process nevertheless met the standards of a fair and objective review.

## Abbreviations

AUC: Area under the curve
BLASTX: Basic Local Alignment Tool
C08: Control of CD experiment in 2008
CD: Controlled deterioration
CD08: Controlled deterioration experiment in 2008
DArT: Diversity Array Technology
DH: Double haploid
E: Expect Value
EPPO: Elevated partial pressure of oxygen
EST: Expressed sequence tag
LOD: Logarithm of odds
OWB: Oregon Wolfe Barley
“OWB-D”: Dominant parent Dom
“OWB-R”: Recessive parent Rec
P50: Half-viability period
QTL: Quantitative trait locus
R^2^: Explained phenotypic variation
RH: Relative humidity
ROS: Reactive oxygen species
TC: Treatment Control of EPPO experiment in 2011
T50: Time to 50% germination
%NS: Percentage of normal seedlings
%TG: Percentage of total germination.
