# Supplementary material for: Barley Seed Aging: Genetics behind the Dry Elevated Pressure of Oxygen Aging and Moist Controlled Deterioration
Source: Front Plant Sci. 2016 Mar 31;7:388. doi: 10.3389/fpls.2016.00388 (PMC4814755; doi:10.3389/fpls.2016.00388)
Supplement: Supplementary file 1 [file DataSheet1.docx]

Supplementary Material

Barley seed ageing: genetics behind the dry elevated pressure of oxygen ageing and moist controlled deterioration

**Manuela Nagel1*, Jan Kodde2, Sibylle Pistrick1, Martin Mascher1, Andreas Börner1 and Steven P. C. Groot2***

**Correspondence: Manuela Nagel, Nagel@ipk-gatersleben.de**

# Supplementary Figures and Tables

**Supplementary Figure 1.**Pre-experimental evaluation of EPPO treatment duration. Ten lines of the ‘W766’ barley population multiplied and stored together with seeds of the OWB population were kept at 40% relative humidity and 20°C and treated for 4, 6, 8, 12 weeks in oxygen at 18 MPa (atmospheric pressure is about 0.1 MPa) and for 12 weeks at 0.2 MPa (normal atmospheric pressure). Germination performance is expressed as percentage of normal seedlings (NS%), total germination (TG%), area under the curve (AUC), time to 50% germination (T50 in h). Boxplots show 25% and 75% quartile, median and whiskers of 1.5 interquartile range for the ten tested lines.


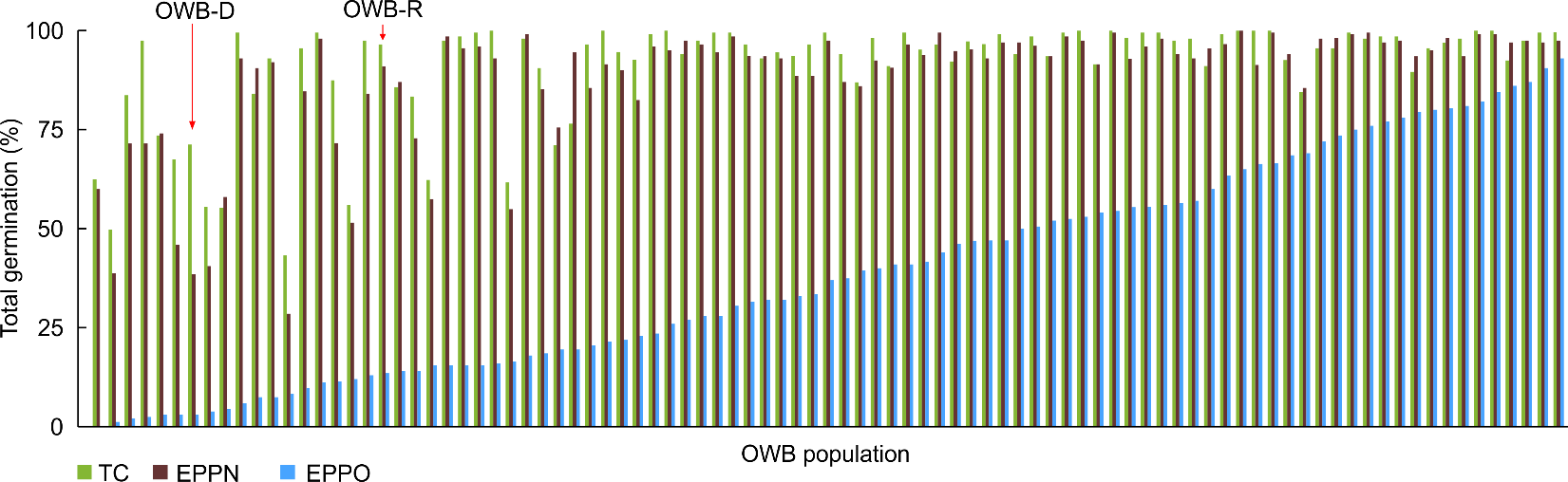


**Supplementary Figure 2.** Total germination of Oregon Wolfe Barley (OWB) lines after different storage treatments. TC, treatment control at ambient air pressure; EPPN, elevated partial pressure of nitrogen storage; EPPO, elevated partial pressure of oxygen storage; “OWB-D”, dominant parent; “OWB-R”, recessive parent.

**Supplementary Table 1.** Effect of different storage treatments on seed germination of Oregon Wolfe Barley (OWB) population. Population mean, standard deviation (SD), minimum (min) and maximum (max) was analysed on basis of 93 OWB lines, mean and SD of dominant parent (“OWB-D”) and recessive parent (“OWB-R”) on basis of four replicates. C08, control performed in 2008; CD08, controlled deterioration performed in 2008; EPPO, elevated partial pressure of nitrogen storage; EPPO, elevated partial pressure of oxygen storage; TC, treatment control at ambient air pressure; %NS, percentage of normal seedlings; %TG, percentage of total germination; T50, time to reach 50% of germinated seeds (h); AUC, area under the curve; P50, half-viability period (d); a, b, c, and d symbolize significant differences between treatments at *P < 0.05*.

| **Trait** | **Treatment** | **Missing** | **‘OWB-D’** | **‘OWB-R’** | | **Mean** |  | **SD** | **Max** | | **Min** | |  |
| --- | --- | --- | --- | --- | --- | --- | --- | --- | --- | --- | --- | --- | --- |
| **%NS** | **TC** | 0 | 54.0 | | 92.5 | 86.2^a^ |  | 16.3 | | 99.5 | | 33.8 | |
|  | **EPPN** | 0 | 66.5 | | 88.0 | 80.9^b^ |  | 18.9 | | 98.5 | | 20.3 | |
|  | **EPPO** | 1 | 1.0 | | 3.0 | 19.1^c^ |  | 19.2 | | 73.5 | | 0.0 | |
|  | **C08** | 0 | 77.0 | | 96.0 | 77.2^b^ |  | 18.3 | | 98.0 | | 12.0 | |
|  | **CD08** | 0 | 42.6 | | 40.5 | 44.3^d^ |  | 24.7 | | 89.5 | | 1.5 | |
| **%TG** | **TC** | 0 | 73.5 | | 100.0 | 90.7^a^ |  | 13.7 | | 100.0 | | 43.3 | |
|  | **EPPN** | 0 | 74.0 | | 93.0 | 86.9^a^ |  | 16.9 | | 100.0 | | 28.5 | |
|  | **EPPO** | 1 | 3.0 | | 16.0 | 38.9^b^ |  | 27.0 | | 93.0 | | 0.0 | |
|  | **C08** | 0 | 87.5 | | 99.5 | 88.7^c^ |  | 15.2 | | 100.0 | | 17.0 | |
|  | **CD08** | 0 | 62.6 | | 66.8 | 60.3^d^ |  | 28.5 | | 97.1 | | 2.4 | |
| **AUC** | **TC** | 0 | 35.8 | | 72.3 | 63.0^a^ |  | 14.1 | | 87.7 | | 23.7 | |
|  | **EPPN** | 0 | 40.8 | | 67.4 | 59.5^a^ |  | 15.8 | | 85.7 | | 16.6 | |
|  | **EPPO** | 3 | 1.1 | | 2.5 | 13.9^b^ |  | 11.9 | | 46.0 | | 0.1 | |
| **T50** | **TC** | 0 | 49.2 | | 27.6 | 30.8^a^ |  | 7.5 | | 51.0 | | 12.0 | |
|  | **EPPN** | 0 | 43.5 | | 26.8 | 31.7^a^ |  | 7.5 | | 49.4 | | 12.1 | |
|  | **EPPO** | 3 | 62.5 | | 88.6 | 69.1^b^ |  | 13.3 | | 116.7 | | 43.4 | |
| **P50** | **TC** | 3 | 173.0 | | 5089.1 | 2233,8 |  | 2165.3 | | 241976.3 | | -19185.7 | |
|  | **EPPN** | 0 | -193.4 | | 238.2 | -209.8 |  | 2673.1 | | 3446.6 | | -19084.8 | |
|  | **EPPO** | 0 | 2.6 | | 27.3 | 38.5^a^ |  | 21.8 | | 111.8 | | -17.5 | |
|  | **CD08** | 3 | 2.4 | | 2.6 | 2.3^b^ |  | 3.5 | | 12.3 | | -11.2 | |

**Suppl. Table S2**

**Functional annotation of genes detected within QTL regions.** Sequences of flanking markers of QTL regions were mapped to the whole-genome shotgun assembly of barley cv. ‘Morex’. Primary alignments with mapping quality ≥ 30 were extracted. Genetic positions of QTL regions in the POPSEQ genetic map (Mascher et al., 2013) were determined from the alignments of marker sequences to the Morex WGS contigs anchored by POPSEQ and functional annotation of all genes listed.
